# Effects of metformin on the glucose regulation, lipid levels and gut microbiota in high-fat diet with streptozotocin induced type 2 diabetes mellitus rats

**DOI:** 10.1007/s12020-024-03843-y

**Published:** 2024-05-23

**Authors:** Xuan Zhou, Jian Zhou, Qingfeng Ban, Mei Zhang, Bo Ban

**Affiliations:** 1 Department of Intensive Care Unit, Affiliated Hospital of Jining Medical University, Jining Medical University, Jining, China; 2 Department of Endocrinology, Genetics and Metabolism, Affiliated Hospital of Jining Medical University, Jining Medical University, Jining, China

**Keywords:** Metformin, Blood glucose, Serum lipids, Gut microbiota, Specific probiotics, High-fat diet with streptozotocin induced

## Abstract

**Objective:**

Metformin, an anti-diabetic drug, regulates blood glucose by affecting gut microbiotas. However, the potential mechanism underlying this effect remains unclear. This study aimed to evaluate the effect of metformin on glucose regulation, lipid levels, and the gut microbiota in rats with type 2 diabetes mellitus induced by a high-fat diet with streptozotocin.

**Research design methods:**

Thirty Wistar rats was using in this experiment. T2DM rats were administered 300 mg/kg metformin for 8 weeks. The glucose regulation, lipid levels, organ coefficients, and gut microbiotawere measured by 16S rDNA.

**Result:**

The metformin-gavaged rats exhibited significant improvements in blood glucose and serum lipid levels, accompanied by alterations in short-chain fatty acid levels and the intestinal microbiota (*p* < 0.05). In the diabetic rats, metformin potentially increased specific probiotics, thus improving the hypoglycaemic effects of the oral anti-diabetic drug. Further, damage to the liver and kidney was effectively alleviated in the metformin-gavaged rats.

**Conclusion:**

This study’s findings demonstrate that metformin exerts a positive anti-diabetic effect in HFD- and STZ-induced T2DM rats. These findings potentially provide a basis for the recommended use of metformin as a reliable oral drug for T2DM owing to its positive effect on the intestinal microbiota.

## Introduction

The recent rise in high-salt, high-fat, and high-sugar diets has led to a gradual annual increase in the incidence of diabetes, posing a threat to human health [1]. Type 2 diabetes mellitus (T2DM) accounts for 95% of all diabetes cases. The number of adults with T2DM has been estimated to reach 700 million by 2045 [1]. The prevalence of T2DM among Chinese adults was 10.4% in 2023 [2]. Complications arising from poorly controlled blood sugar levels in T2DM can adversely impact patients’ health and impose a profound economic burden on affected individuals. In the United States, healthcare expenditure on diabetes accounts for approximately 14% of the total healthcare expenditure, much of which is related to diabetes-related complications, such as myocardial infarction and stroke [3]. Unsurprisingly, relevant surveys conducted in China yielded similar findings wherein patients with a disease duration >10 years incurred nearly three-fold higher healthcare expenditure than those with a disease duration <5 years [2]. An individual’s innate genetic differences and acquired environmental influences are generally considered to play a significant role in the pathogenesis of T2DM [4]. The combination of these factors potentially results in the relative or absolute absence of secretory function in pancreatic β-cells, increased liver glycogenesis, and decreased glucose uptake in muscle and liver tissues. Other factors include the decreased secretion of certain glucose-regulating hormones in the intestines, increased glucose reabsorption in the kidneys, abnormalities in neurotransmitter secretion, and increased glucagon secretion [5]. Gut microbiotas have recently become a topic of considerable research interest, with a growing body of evidence suggesting that under healthy conditions, a dynamic balance is maintained in the gut microecology, whereas the disruption of this homoeostasis can alter the composition and function of the gut microbiota, leading to disease [6–8]. Numerous studies have reported a special environmental factor involved in the pathogenesis of T2DM, namely, the microbial community colonising our digestive tract [9]. Current treatment methods for T2DM mainly involve oral antihyperglycaemic drugs [10]. However, these drugs are prone to adverse reactions (hypoglycaemia and weight gain), increase the risk of cardiovascular disease, and exhibit poor efficacy [11, 12]. Metformin, a biguanide drug, is the most popular oral antihyperglycaemic medication used in most countries and is widely considered the basic therapy for newly diagnosed patients with T2DM [13, 14].

Its reputation predominantly stems from its effective glucose-lowering ability, low cost, weight-neutral effects, favourable overall safety (especially the absence of hypoglycaemia-related side effects), and moderate evidence for cardio-protection [15]. Metformin is a guanidine derivative originally extracted from French lilac or goat’s rue (*Galega officinalis*). Metformin was first synthesised in 1922 and introduced into the human body as a medication by Jean Sterne in 1957 [16]. Its efficacy in treating T2DM has been demonstrated alone and in combination with other antihyperglycaemic drugs. Owing to these key characteristics, extensive interest in this drug continues to persist several decades after its inclusion in the diabetes pharmacopeia. The oral bioavailability of metformin is approximately 30–60%, its intestinal concentration is 100–300 times higher than its serum concentration, and approximately 50% of the ingested dose is present in stool. When taken orally, metformin has a half-life of approximately 3–4 h, exhibiting inconsistency with its hypoglycaemic effects [15, 16].

With recent advances in detection technologies, knowledge regarding the gut microbiota’s important role in the pathogeneses of obesity and diabetes is increasing. Research has increasingly focused on the relationship between metformin and the gut microbiota [17–19]. Nevertheless, owing to inconsistencies in research findings, the mechanism by which metformin affects the gut microbiota remains unclear. Therefore, this study aimed to examine the effects of metformin on glucose regulation, lipid levels, and the gut microbiota in rats with T2DM induced by a high-fat diet (HFD) with streptozotocin (STZ). The findings may serve as a reference for understanding the effects of metformin on the gut microbiota.

## Materials and methods

### Materials

Metformin and STZ were purchased from Sigma-Aldrich (St. Louis, Missouri, USA). A pelletised high-fat diet (60% kcal fat) (D12492) was purchased from Trophic Animal Feed High Technology Co., Ltd (Nantong, China). Unless otherwise indicated, all other chemicals used in the current study were of analytical grade.

### Animal models

The animal experiment was performed at the National Animal Feeding Engineering Center, Jiangnan University, in accordance with the ‘Animal Testing Guidelines’ established by the Committee for the Care and Use of Laboratory Animals, Jiangnan University [(SYXK (SU) 2016-0045), approval number: JN.No20180915W0801120 (194)].

Thirty Wistar rats (5 weeks old, male, no specific pathogen grade) were purchased from the Shanghai Slake Animal Center and divided into three groups based on body weight (Table 1). Group 1 comprised 10 rats (control group, CT) that were fed a normal diet and allowed to adapt to their new environment. On the first day of week 6, the rats were administered an intraperitoneal injection of 50 mmol L^–1^ citrate buffer (pH 4.5). The CT rats were fed a normal diet and gavaged with 50 mg mL^–1^ distilled water per kilogram of body weight (BW) from week 2 to week 7. Groups 2 (diabetes control group, T2DM) and 3 (metformin group, METFM) also included ten rats each. Like those in the CT group, the rats in groups 2 and 3 acclimated to their new environment during the first week. From week 2 to week 7, both groups underwent HFD feeding. On the first day of week 6, the twenty rats in groups 2 and 3 were administered STZ dissolved in 50 mmol L^–1^ citrate buffer (pH 4.5) via intraperitoneal injection at a dose of 100 mg kg^–1^ BW. From week 2 to week 7, the T2DM group was provided 30 mg mL^–1^ distilled water once daily, while the METFM group was administered 300 mg kg^–1^ BW metformin once daily. On day 7 after STZ injection, rats with blood glucose levels >10.0 mmol L^–1^ were identified as diabetic models. Their blood was centrifuged using an ultra-low temperature centrifuge for 15 min (4 °C, 3000 × *g*), and the upper liquid, which was the serum, was carefully absorbed using a pipette gun and frozen in an ultra-low temperature refrigerator at –80 °C for later use.

**Table 1:** Experiment design

	Oral administration					STZ injection		
	1	2	3	4	5	6	7	
CT	Adapt to their new environment	Normal diet and 50 mg mL^−1^ distilled water						Kill and sampling
T2DM		High fat diet and 50 mg mL^−1^ distilled water						
METFM		High fat diet and 300 mg kg^−1^ metformin						

### Monitoring of the rats’ metabolic status using a Clinical Laboratory Animal Monitoring System

On experimental day 42, the rats were placed in a metabolic chamber for self-regulation. Their metabolic rate was monitored every 13 min for the subsequent 24 h. The heat level was also closely monitored throughout the measurement period [20].

### Oral Glucose Tolerance Test (OGTT)

The OGTT followed procedures described in previous reports [21]. Blood glucose levels were measured 0, 30, 60, 90, and 120 min after glucose administration using an ultra-blood glucose metre (Life Scan, Milpitas, NJ, USA) via the tail vein.

### Biochemical analysis

Serum total triglyceride (TG), total cholesterol (TC), low-density lipoprotein cholesterol (LDL-C), high-density lipoprotein cholesterol (HDL-C), insulin, and glycated haemoglobin (HbA1c) levels were quantified using a commercial kit (Nanjing Jiancheng Biotechnology Co., Ltd., Nanjing, China) [22].

### Measurement of organ coefficients

Liver, kidney, spleen, and thymus tissues were immediately removed and rinsed with ice-cold 0.85% saline solution after scarification of the rats. Thereafter, tissue capsules were carefully removed and weighed promptly after euthanisation.

### Pancreatic histological analysis

The pancreas of each rat was excised, preserved in 4% paraformaldehyde solution, embedded, and sliced. Subsequently, the sections were dewaxed and stained with haematoxylin eosin (H&E). The morphology of the pancreatic sections was examined using an Olympus IX83 microscope (Tokyo, Japan) [22].

### Faecal analyses of short-chain fatty acid (SCFA) content and gut microbiota

Rat faeces were collected 1 day before the end of the study period. The four major faecal SCFAs, namely, acetic acid, propionic acid, butyric acid, and valeric acid, were measured in all groups. Each faecal sample (0.2 g) was placed in a 15-mL centrifuge tube, and 10 mL of water was added to dissolve the sample. The upper ether solution was analysed via gas chromatography using a 30 m × 0.25 × 0.25 μm ‘Free Fatty Acid Phase’ elastic quartz capillary column at 100 °C for 1 min, increasing by 5 °C/min for 5 min to 150 °C [23].

Microbial DNA was extracted from faecal samples, and the 16S rRNA gene for isolated DNA was sequenced according to the manufacturer’s guidelines using the Illumina MiSeq 2500 platform (a service provided by gene DENOVO, Inc.). Thereafter, 16S rRNA gene sequence de-multiplexing, quality control, and operational taxon unit partitioning were performed using a standard pipeline with Mothur software (version 1.3.4.0). Statistical tests were performed in R, a free tool for scientific computing [24].

### Statistical analysis

Data are expressed as the mean ± standard deviation of six rats per group. All data were analysed using SPSS 20.0 (SPSS, Inc., Chicago, IL, USA). Univariate analysis of variance and Duncan’s multi-range test were used to evaluate differences between groups. Statistical significance was set at *p* < 0.05.

## Results and discussion

### Body weight, food intake, and water intake

**Fig. 1:**
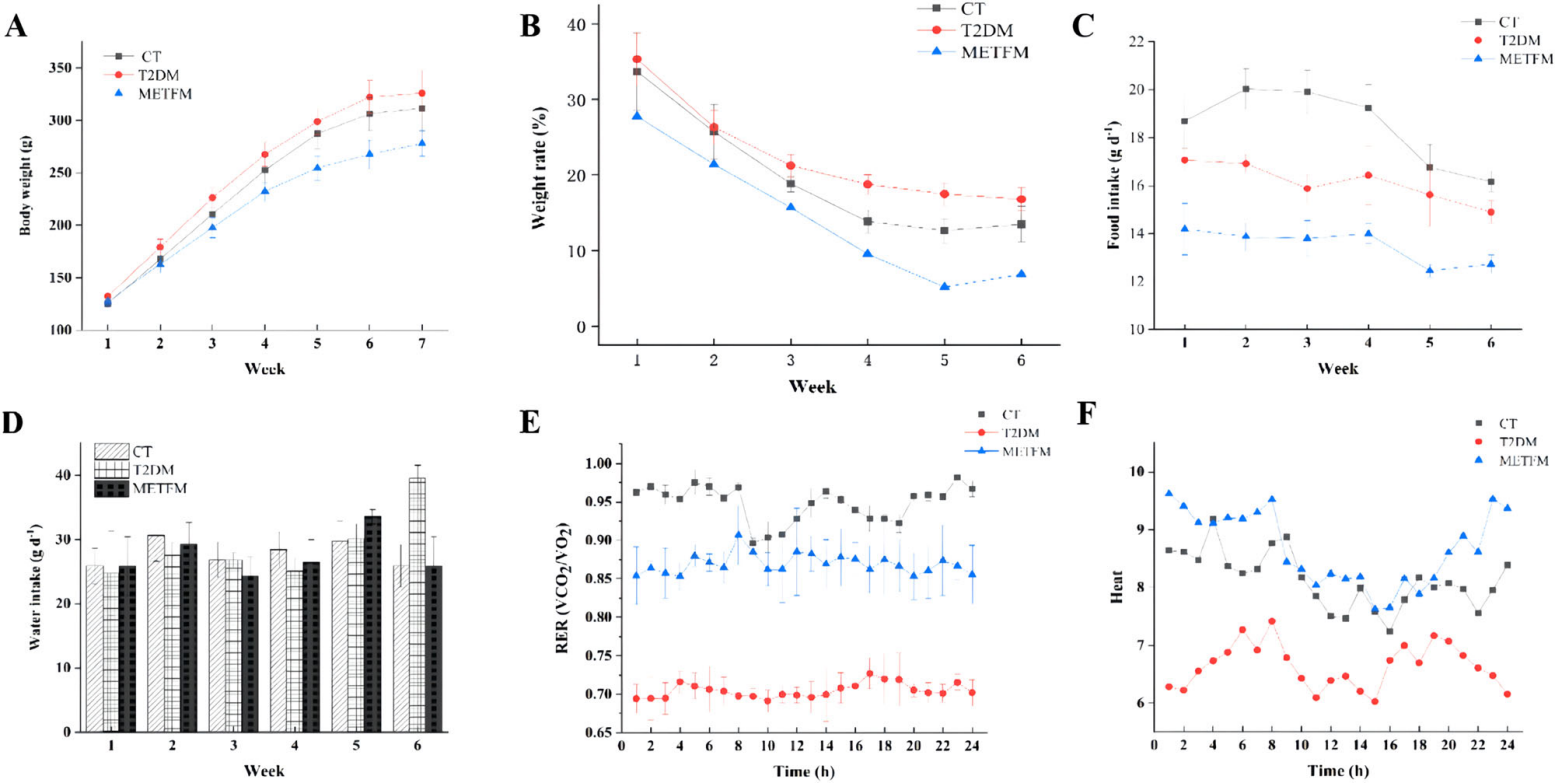
Effect of metformin on the body weight, food intake, RER rate, and heat level of diabetic rats. Note: **A** body weight; **B** weight rate of each group; **C** food intake; **D** water intake; **E** RER level; **F** heat level; CT control group; T2DM group: the rats fed with high fat diet; METFM: the rats fed with high fat diet and metformin

During the experiment, rat body weight was monitored in all groups (Fig. 1A, B). In week 1, the initial weights of all rats were similar (*p* > 0.05); however, after week 2, the body weights of the T2DM group exceeded those of the other two groups, with significant differences (*p* < 0.05). This indicates that HFD feeding affects the metabolism of T2DM rats, leading to weight gain. By week 6, the METFM group’s body weights were lower than those of the other two groups, with significant differences (*p* < 0.05), implying that metformin intervention potentially prevents HFD-induced obesity. After 6 weeks of treatment, the body weights of METFM rats had decreased by 14.76% compared with those of T2DM rats. This also suggests that 6 weeks of metformin treatment effectively mitigates the effects of HFD feeding on rat body weight. Our findings are consistent with the clinical results obtained by Golay [25] wherein metformin intervention significantly reduced the body weights of patients with T2DM.

As shown in Fig. 1C, food and water intake in the METFM group was significantly suppressed after metformin intervention (*p* < 0.05). During the first 4 weeks, no fluctuations were observed in the T2DM and METFM groups. By week 5, food and water intake in these two groups had exhibited a downward trend compared with that in the CT group. After STZ administration in week 6, food and water intake increased. These results demonstrate that the HFD-fed groups reduced their food intake as a form of stress protection from the start of the experiment to week 5. However, STZ administration destroyed their pancreatic β-cells and caused an imbalance in glucose and lipid metabolism, resulting in increased food and water intake. During the first 5 weeks of the experiment, water intake in the T2DM group did not differ significantly (*p* > 0.05) from that in the CT group. These results suggest that METFM group rats mitigated HFD- and STZ-induced damage by reducing food intake and increasing water intake, ultimately affecting their body weight [26].

### Respiratory exchange rate (RER) and heat level

An RER value approximating 1.0 indicates the use of carbohydrates as the main metabolic substrate by experimental animals. When the RER value approaches 0.7, fat becomes the predominant metabolic substrate [27]. As shown in Fig. 1E, the CT group’s RER value approximated 1.0, demonstrating that it used carbohydrates as the main energy source during the 42-day experiment. The RER value of the T2DM group decreased compared with that of the CT group. The RER value of the T2DM group approached 0.7, indicating that the main energy source of the rats in this group was fat (*p* < 0.05). Compared with the T2DM group, the METFM group displayed a greater improvement in the RER value (*p* < 0.05), which approximated 0.9, suggesting that metformin potentially alleviates obesity and other HFD-induced side effects by resolving metabolic abnormalities in T2DM rats. The heat level reflects the spontaneous activity of the rats within 24 h. As shown in Fig. 1F, HFD feeding decreased the heat levels of the T2DM group compared with those of the CT group, resulting in fat accumulation. After 42 days of metformin intervention, the heat levels of the METFM group were significantly higher than those of the T2DM group (*p* < 0.05), indicating that metformin potentially alleviates T2DM-related symptoms by increasing the rats’ spontaneous activity.

### Fasting blood glucose (FBG) levels

**Fig. 2:**
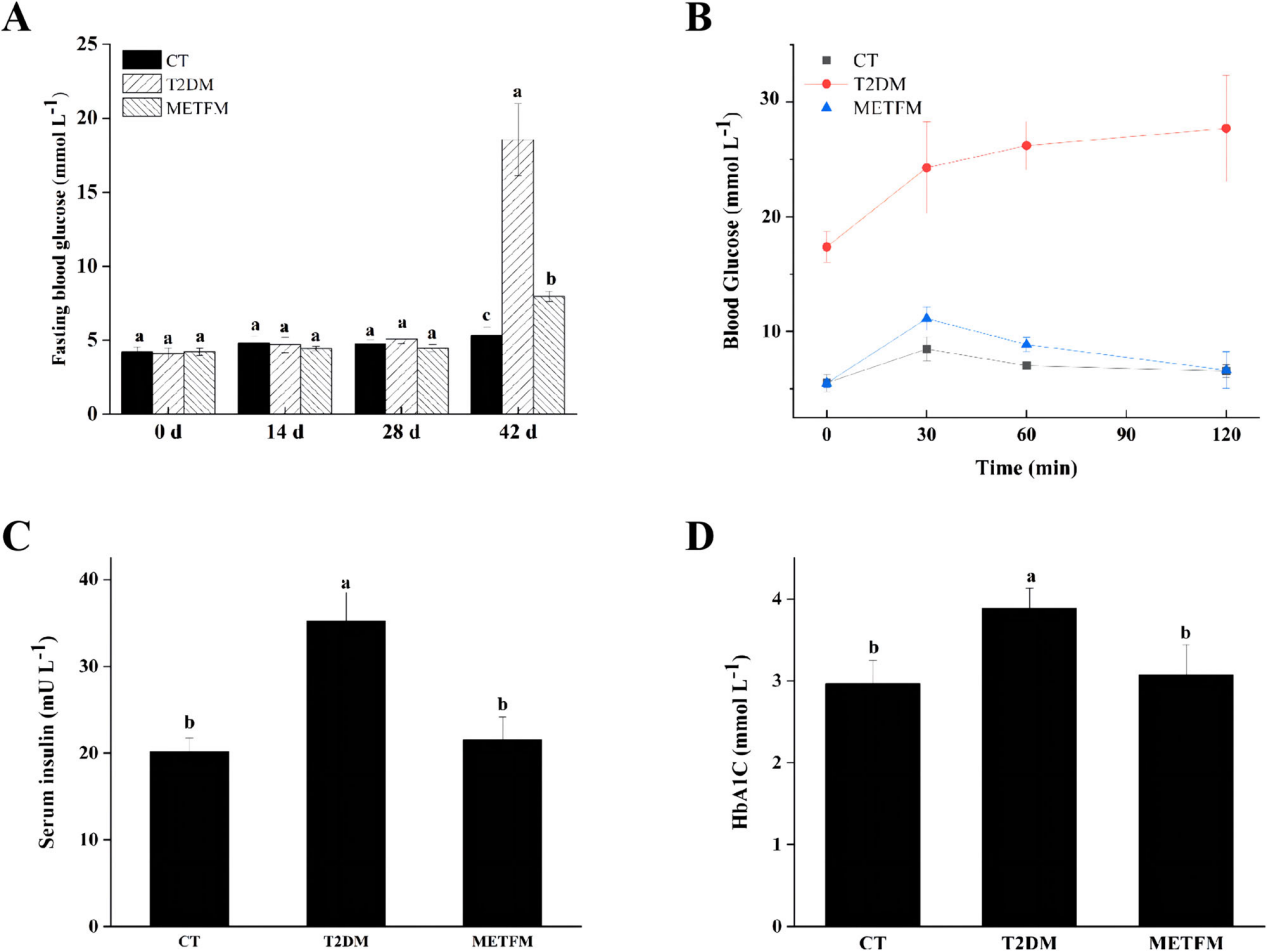
Effect of metformin on the fasting blood glucose, OGTT, Serum insulin, and HbA1C levels Note: **A** fasting blood glucose; **B** OGTT; **C** serum insulin; **D** HbA1C level; CT control group; T2DM group: the rats fed with high fat diet; METFM: the rats fed with high fat diet and metformin; Different lower case letters denote significance differences between groups treated at *P* < 0.05

Long-term hyperglycaemia may lead to tissue damage and pathological changes in the organs, leading to diabetic complications. Strict blood glucose control can help mitigate diabetic complications, including microvascular and cardiovascular diseases [28]. The FBG levels of each group were measured once every 2 weeks. Figure 2A reveals no significant differences in FBG among the different groups during the first 28 days (*p* > 0.05), suggesting that short-term HFD feeding exerts a minimal effect on the rats’ FBG levels. By week 7, the T2DM group’s FBG level significantly differed from that of the CT group (*p* < 0.05), indicating that intraperitoneal SZT injection in T2DM rats led to the destruction of their pancreatic islet β-cells, thus increasing FBG levels. The METFM group’s FBG level was significantly lower than that of the T2DM group (*p* < 0.05). Therefore, metformin may improve the FBG levels of T2DM rats and alleviate HFD- and STZ-induced hyperglycaemia in T2DM rats [29].

### OGTT

The OGTT is the most widely used means of detecting diabetes. It is a glucose load test that evaluates the function of pancreatic β-cells and the body’s ability to regulate blood glucose [30]. The OGTT results of each group after 42 days are presented in Fig. 2B. The blood glucose levels of each group increased rapidly after gavaging with glucose, peaking at 30 min in the CT and METFM groups. The blood glucose levels of the CT and METFM groups returned to normal at 120 min. The OGTT results of the diabetic rats in the METFM group significantly improved after 42 days of metformin intervention. However, the blood glucose levels of T2DM rats remained relatively high. Therefore, metformin may effectively improve the OGTT results of T2DM rats. These results are consistent with those of FBG, suggesting that metformin potentially reduces FBG levels by enhancing glucose tolerance [31].

### Serum insulin and HbA1c levels

Insulin, a protein hormone secreted by pancreatic β-cells, regulates the body’s glucose metabolism and blood glucose homoeostasis [32]. The serum insulin levels of each group after 42 days are shown in Fig. 2C. The serum insulin levels of T2DM rats were significantly higher than those of CT rats (*p* < 0.05), and these levels changed significantly after metformin intervention (*p* < 0.05). Compared with those of the T2DM group, the METFM group’s serum insulin levels decreased by 38.94%. HbA1c, a form of haemoglobin, is non-enzymatically linked to glucose and represents the average blood glucose concentration over 4 or 12 weeks. It is one of the key indicators of long-term blood glucose levels [33]. The HbA1c levels of all groups after 42 days are shown in Fig. 2D. The HbA1c levels of the T2DM group markedly increased (*p* < 0.05), whereas those of the METFM group decreased, exhibiting a positive effect (*p* < 0.05). This suggests that metformin improves serum HbA1c levels.

### Serum lipid levels

Hyperglycaemia is closely associated with serum dyslipidemia, a major cause of T2DM-related complications [34]. Compared with those of the CT group, the TG, TC, and LDL-C levels of the T2DM group were significantly elevated (*p* < 0.05), whereas the HDL-C level was significantly reduced (*p* < 0.05) (Table 2). Compared with those of the T2DM group, the TG, TC, and LDL-C levels of the METFM group were markedly reduced (*p* < 0.05), whereas the HDL-C level was significantly elevated (*p* < 0.05). Therefore, metformin helps improve T2DM rat serum lipid levels [35].

**Table 2:** Effect of metformin on the serum total lipids levels in the diabetic rats

Groups	Total lipids			
	TG	TC	HDL-C	LDL-C
Con	2.51 ± 0.58^a^	2.14 ± 0.21^a^	1.41 ± 0.14^b^	0.45 ± 0.07^a^
T2DM	4.12 ± 0.42^b^	3.59 ± 0.41^c^	0.98 ± 0.13^a^	0.71 ± 0.16^b^
METFM	3.12 ± 0.22^b^	2.54 ± 0.25^b^	1.23 ± 0.21^ab^	0.47 ± 0.11^ab^

Different lower case letters denote significance differences between groups treated at *P* < 0.05

### Organ coefficients

The organ-weight-to-body-weight ratio remains relatively constant under normal conditions. However, when an animal is diseased or in suboptimal health, the weight of a damaged organ may undergo pathological changes, leading to corresponding changes in the organ coefficient [36]. The organ coefficients of the groups after 42 days are shown in Table 3. Significant differences in the liver and kidney coefficients were noted between the CT and T2DM groups (*p* < 0.05). Nonetheless, the impact on the thymus and spleen was not significant (*p* > 0.05). This indicates that long-term HFD feeding potentially causes liver and kidney damage in diabetic rat models. The liver and kidney coefficients of the METFM group were markedly lower than those of the T2DM group (*p* < 0.05). However, the thymus and spleen remained unaffected (*p* > 0.05). The METFM group exhibited 12.30% and 11.71% decreases in its liver and kidney coefficients, respectively, compared with the T2DM group.

**Table 3:** Effect of metformin on the organ coefficients in the diabetic rats

Groups	Organ coefficients (mg g^−1^ BW)			
	Liver	Thymus	Spleen	Kidney
Con	37.31 ± 6.35^a^	1.12 ± 0.31^a^	1.92 ± 0.23^a^	6.07 ± 0.65^a^
T2DM	57.65 ± 3.93^c^	1.24 ± 0.25^a^	1.97 ± 0.17^a^	8.11 ± 0.48^b^
METFM	50.56 ± 2.22^b^	1.28 ± 0.23^a^	2.18 ± 0.13^a^	7.16 ± 0.37^ab^

Different lower case letters denote significance differences between groups treated at *P* < 0.05

### Pancreatic tissue changes

**Fig. 3:**
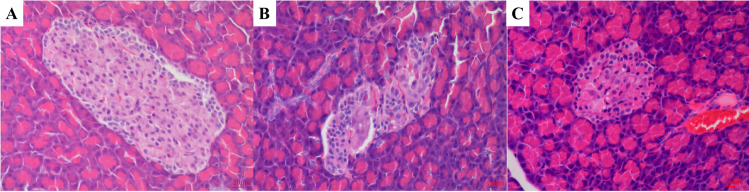
The H&E image of panreas. Note: **A** control group (CT); **B** the rats fed with high fat diet (T2DM group); **C** the rats fed with high fat diet and metformin (METFM)

The H&E sections of pancreatic tissues from each group are shown in Fig. 3A–C. Differences in the overall morphology and number of islets of Langerhans in the pancreatic tissues were observed across the different groups. In the CT group (Fig. 3A), the islets of Langerhans in the pancreatic tissues were round and large, with clear boundaries and intact pancreatic acinar cells. In the T2DM group, the islets of Langerhans were significantly atrophied, with unclear boundaries and a diminished number of pancreatic acinar cells (Fig. 3B). Although the pancreatic tissues exhibited smaller islet areas and irregular islet morphology after 42 days of metformin intervention, the number of core cells increased, indicating that metformin can alleviate HFD- and STZ-induced damage to pancreatic tissues (Fig. 3C). Nna et al. investigated the synergistic effects of Malaysian propolis and metformin on HFD- and STZ-induced diabetic rats and found that a combination of both could effectively improve the islets of Langerhans in pancreatic tissues [37].

### Gut microbiota and SCFA levels

Alpha diversity (α-diversity) refers to diversity within a specified area and is commonly measured using a series of indexes, including the Chao1, Ace, Simpson, and Shannon indexes [38]. Information regarding species diversity can be obtained through diversity analysis. In this study, we employed the Chao1, Ace, Simpson, and Shannon indexes to assess the effects of metformin on the gut microbiota’s α-diversity in HFD- and STZ-induced T2DM rats. The Chao1 and Ace indexes were used to assess species richness, while the Simpson and Shannon indexes were used to assess the distribution of species diversity [39]. S-Table 1 shows that the HFD- and STZ-induced T2DM group yielded lower Chao1 and Ace indexes than the other groups. Compared with that of the T2DM group, the Chao1 index of the METFM group significantly increased (*p* < 0.05), suggesting that metformin facilitates the recovery of the gut microbiota’s α-diversity. However, the METFM group generated lower Chao1 and Ace indexes than the CT group. These results imply that oral gavaging with metformin somewhat improved the gut microbiota diversity of T2DM rats but failed to achieve the same level of diversity as that of healthy rats. The Simpson and Shannon indexes were both used to estimate the microbial diversity of the sample; however, they were calculated using different methods. A high Simpson index indicates poor community diversity, whereas a high Shannon index indicates rich community diversity. S-Table 1 reveals that the HFD- and STZ-induced T2DM group had higher Simpson and lower Shannon indexes than the other groups. The METFM group yielded significantly increased Shannon and decreased Simpson indexes (*p* < 0.05) compared with the T2DM group. These findings suggest that long-term HFD feeding combined with STZ treatment decreased the α-diversity of the gut microbiota in rats; however, metformin intervention facilitated its recovery.

**Fig. 4:**
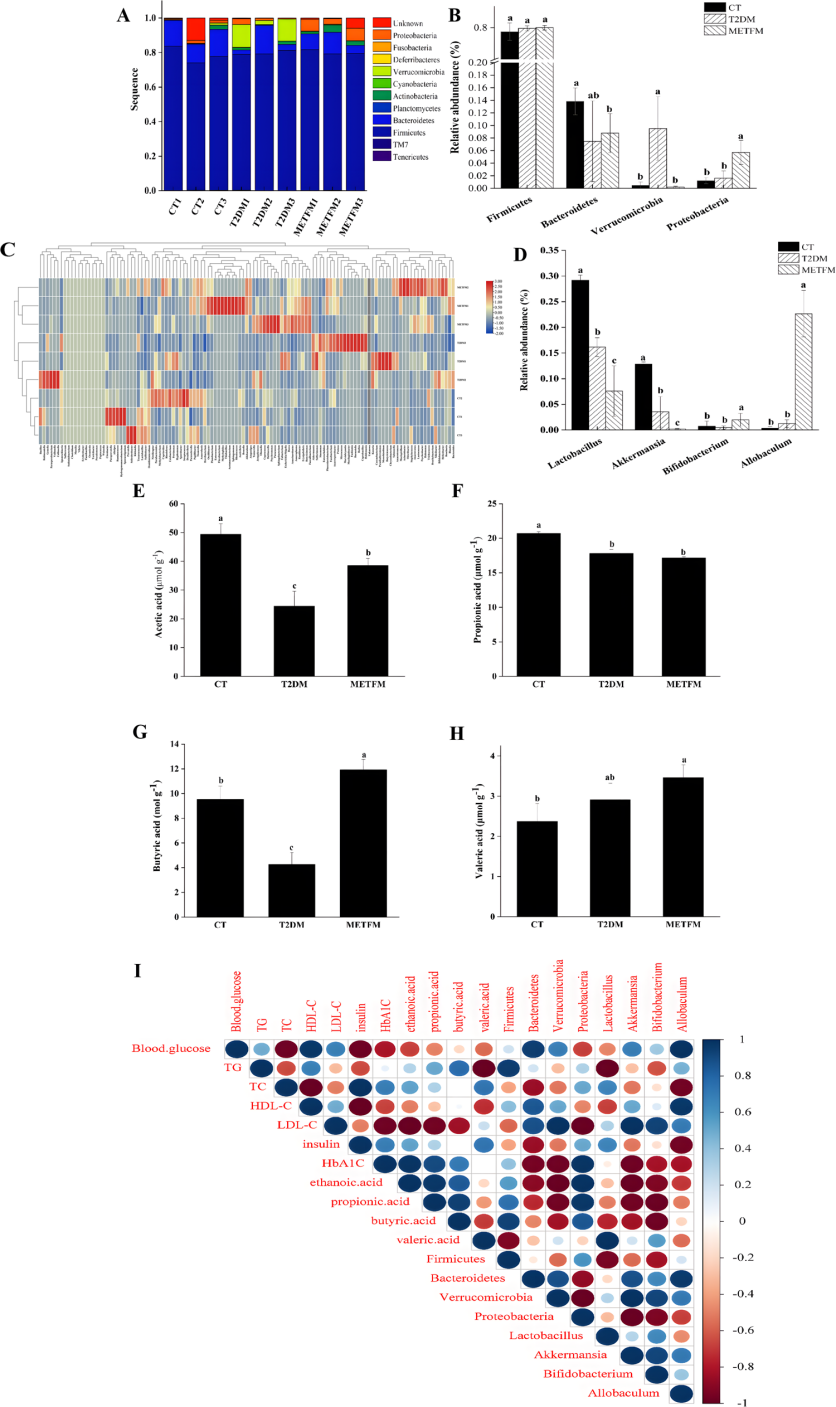
Effect of metformin on the gut microbiota and SCFAs levels. Note: **A** heatmap of phylum level; **B** the abundance of phylum level; **C** heatmap of genus levels; **D** the abundance of genus level; **E** ethanoic acid; **F** propionic acid; **G** butyric acid; **H** valeric acid; **I** the statistical correlation analysis. Different lower case letters denote significance differences between groups treated at *P* < 0.05

Patients with T2DM suffer from dysbiosis, which is reflected by an increase in pathogenic microorganisms. High-throughput 16S rRNA sequencing is often employed to analyse microbial composition. At the phylum level, the microbial communities in all rat faecal samples chiefly included *Firmicutes*, *Bacteroidetes*, *Actinobacteria*, *Cyanobacteria*, *Deferribacteres*, *Proteobacteria*, *TM7*, *Tenericutes*, and *Verrucomicrobia* (Fig. 4A). Comparisons between the CT and T2DM groups indicated that the abundances of *Bacteroidetes* and *Verrucomicrobia* differed significantly (*p* < 0.05), whereas those of *Firmicutes* and *Proteobacteria* did not (*p* > 0.05). After metformin intervention, the three most abundant phyla underwent changes (*p* < 0.05) (Fig. 4B). Metformin treatment significantly increased the level of *Proteobacteria* but negatively affected *Bacteroidetes* and *Verrucomicrobia* levels, suggesting that metformin has targeted proliferative effects on *Proteobacteria*. Molina-Vega et al. also reported that *Proteobacteria* abundance increased in the METFM group compared with that in the INS groups [40].

A total of 119 phenotypes were isolated from the gut microbiota as key variables to determine how they are affected by different treatments at the genus level. Figure 4C reveals significant changes in the heatmap distributions and genus-level relative abundances in all rat faecal samples. The heatmaps indicate that the different treatment groups underwent changes in the microbial community characteristics to varying extents. The T2DM group had significantly decreased *Lactobacillus*, *Akkermansia*, and *Bifidobacterium* levels compared with the CT group (*p* < 0.05) but exhibited no significant difference for *Allobaculum* (*p* > 0.05). The METFM group displayed significantly reduced *Lactobacillus* and *Akkermansia* levels (*p* < 0.05) and increased *Bifidobacterium and Allobaculum* levels (*p* < 0.05) compared with the T2DM group. These results suggest that metformin may exert targeted proliferative effects on *Bifidobacterium* and *Allobaculum*. Moreover, they are consistent with those generated by Zhang et al. [41] suggesting that metformin potentially alters specific probiotics to improve the hypoglycaemic effects of T2DM rats.

Gut microbes can convert indigestible dietary fibre into SCFAs, predominantly ethanoic acid, propionic acid, butyric acid, and valeric acid. HFD feeding altered the SCFA composition, whereas the uptake of fermentation substrates restored ethanoic acid, propionic acid, butyric acid, and valeric acid levels [42]. The caecal levels of these acids after 42 experimental days are shown in Fig. 4E–H. The ethanoic acid, propionic acid, and butyric acid levels in the T2DM group were significantly lower than those in the CT group (*p* < 0.05), while valeric acid displayed an upward trend. However, the difference was not significant (*p* > 0.05). Compared with the T2DM group, the METFM group exhibited significant differences in the levels of ethanoic acid, butyric acid, and valeric acid (*p* < 0.05), but not propionic acid. These findings indicate that metformin significantly improves the intestinal SCFA levels of T2DM rats [43].

To further investigate metformin’s potential mechanism, a statistical correlation analysis of blood glucose, serum insulin, HbA1c, total lipid, gut microbiota, and SCFA levels was performed (Fig. 4I). As shown in Fig. 4I, specific probiotics (*Proteobacteria* and *Allobaculum*) and SCFAs exhibit strong correlations with blood glucose levels. This possibly emanates from the metformin-induced proliferation of *Proteobacteria* and *Allobaculum*, while the increased SCFA levels may be attributed to specific probiotics.

## Conclusions

This study’s findings demonstrate that metformin exerts a positive anti-diabetic effect in HFD- and STZ-induced T2DM rats. The potential mechanism underlying this effect may be linked to FBG regulation, serum lipid improvement, and gut microbiota alterations in T2DM rats. These findings potentially provide a basis for the recommended use of metformin as a reliable oral drug for T2DM owing to its positive effect on the intestinal microbiota.

## Supplementary information

Supplementary information
