# Supplementary material for: Effects of metformin on the glucose regulation, lipid levels and gut microbiota in high-fat diet with streptozotocin induced type 2 diabetes mellitus rats
Source: Endocrine. 2024 May 23;86(1):163–72. doi: 10.1007/s12020-024-03843-y (PMC11445279; doi:10.1007/s12020-024-03843-y)
Supplement: Supplementary file 1 — Supplementary information [file 12020_2024_3843_MOESM1_ESM.docx]

S-Table 1 The α-diversity of gut microbiota

| Groups | α-diversity of gut microbiota | | | |
| --- | --- | --- | --- | --- |
|  | Chao1 | Ace | Shannon | Simpson |
| Con | 757.1±48.56^a^ | 666.69±19.20 | 6.93±0.19 | 0.96±0.02^a^ |
| T2DM | 319.86±40.97^b^ | 381.96±9.91 | 4.55±0.24 | 1.20±0.01^b^ |
| METFM | 442.45±33.73^b^ | 397.28±39.85 | 5.16±0.15 | 0.94±0.01^ab^ |

Note: Different lower case letters denote significance differences between groups treated at *p* < 0.05.
